# Examining the association between socio-demographic factors, catheter use and antibiotic prescribing in Northern Ireland primary care: a cross-sectional multilevel analysis

**DOI:** 10.1017/S0950268822000644

**Published:** 2022-04-21

**Authors:** C. Nugent, N. Q. Verlander, S. Varma, D. T. Bradley, L. Patterson

**Affiliations:** 1 UK Field Epidemiology Training Programme, UK Health Security Agency, London, UK; 2 Health Protection Department, Public Health Agency, Belfast, Northern Ireland; 3 UK Health Security Agency, London, UK; 4 Queens University Belfast, Belfast, Northern Ireland; 5 Health and Social Care Board, Belfast, Northern Ireland

**Keywords:** Primary care, antibiotics, catheters, deprivation, urban-rural, multilevel modelling

## Abstract

Inappropriate use of antibiotics is among the key drivers of antimicrobial resistance (AMR). Antibiotic use in Northern Ireland (NI) is the highest in the UK and approximately 80% is prescribed in primary care. Little information however exists about the patient and prescriber factors driving this. We described the trend in NI primary care total antibiotic prescribing 2010–2019 and conducted a cross-sectional study using a random sample of individuals registered with an NI GP on 1st January 2019. We used multilevel logistic regression to examine how sociodemographic factors and urinary catheter use was associated with the likelihood of being prescribed an antibiotic during 2019, adjusting for clustering at GP practice and GP federation levels. Finite mixture modelling (FMM) was conducted to determine the association between the aforementioned risk factors and quantity of antibiotic prescribed (defined daily doses). The association between age and antibiotic prescription differed by gender. Compared to males 41–50 years, adjusted odds of prescription were higher for males aged 0–10, 11–20 and 51 + years, and females of any age. Catheter use was strongly associated with antibiotic prescription (aOR = 6.82, 95% CI 2.50–18.64). Socioeconomic deprivation and urban/rural settlement were not associated in the multilevel logistic analysis. GP practices and federations accounted for 1.24% and 0.12% of the variation in antibiotic prescribing respectively. FMM showed associations between larger quantities of antibiotics and being older, male and having a catheter. This work described the profile of individuals most likely to receive an antibiotic prescription in NI primary care and identified GP practice as a source of variation; suggesting an opportunity for reduction from effective interventions targeted at both individuals and general practices.

## Introduction

Antimicrobial resistance (AMR) describes the process by which an organism develops resistance to an antibiotic with the result it can no longer be used to treat an infection. In 2016, the O'Neill review [1] highlighted the global threat posed by AMR with an estimated ten million deaths annually at a cost of 100 trillion USD by 2050. Among the key drivers of AMR is the inappropriate use of antibiotics. In the UK, antibiotic use is highest in Northern Ireland (NI) where consumption is more than 50% higher than in England (data from 2018 published by the Public Health Agency, 2020 [2]). Approximately 80% of all prescribing in NI takes place in primary care (including out-of-hours and dental prescribing).

In response to the O'Neill review, the UK government set out an ambition to halve inappropriate antibiotic consumption in the UK by 2020, and the Department of Health, Northern Ireland endorsed this target for NI. The factors influencing antibiotic consumption, however, are complex [3] and the target was adapted to a 15% reduction in total UK antibiotic use by 2024 with a specific target of 25% reduction in primary care [4]. To effectively reduce antibiotic use in primary care in NI, it is necessary to understand who is consuming antibiotics and how prescribing varies across GP practices. However, little information exists.

The association between antibiotic consumption and age is well-described in both the UK and Europe with people in the youngest [5] and oldest age groups most likely to be prescribed an antibiotic [6]. The association with gender is also relatively consistent, with females generally receiving more antibiotics than males [7] the causes of which are multifactorial. In NI specifically, a recent study utilising data from the 2014/15 Health Survey NI replicated these findings with self-reported antibiotic use [8]. There also appears to be a link between antibiotic prescribing and socioeconomic status [7,9], with those in the most deprived populations most likely to receive antibiotics. The association with rural and urban communities is less clear. Some studies report increased consumption in urban communities [10] while others report higher use in rural populations [11].

In addition to socio-demographic factors, we know that antibiotics are used to treat infections and we know the risk of infection can increase in the presence of co-morbidities. One such factor is the use of a urinary catheter. According to the National Institute for Health and Care Excellence (NICE), catheter-associated urinary tract infections (CAUTIs) comprise a large proportion of healthcare-associated infections (HCAIs) [12] and nearly everyone with a catheter in place will have bacteriuria after just one month [13]. Patients with a catheter are also likely to be older and have other comorbidities which may require antibiotic treatment. Understanding these individual factors is important to help direct public health messaging and influence primary care guidance.

As well as individual factors, the clinical responsibility to prescribe antibiotics lies with a person's General Practitioner (GP). We know the decision to prescribe an antibiotic is influenced by several factors including the ability to make diagnostic decisions, particularly with older patients and those in vulnerable groups, managing patient expectations and having knowledge of alternatives, such as delayed prescriptions [3]. This can lead to variation in who gets an antibiotic across the population. In terms of infrastructure, multiple GP's may serve an individual GP practice, of which there are 321 in NI (330 in 2019). In addition, GP practices belong to a collection of fully-incorporated GP-funded ‘federations’, of which there are 17 in NI. These federations aim to support and protect GP practices and help deliver the transformation agenda in health and social care [14]. We, therefore, have individuals clustered within a GP practice, which are clustered within a GP Federation. Ignoring this clustering can result in incorrect inference of the likely association between individual and GP factors and the odds of being prescribed an antibiotic. This analysis will account for this clustering using multilevel modelling to derive more accurate and valid estimates of the effect.

The study objectives are to; (1) describe trends in primary care antibiotic prescribing for all antibiotics, to establish a baseline against which the impact of interventions can be assessed and; (2) understand the epidemiology of antibiotic prescribing in primary care, with a view to potentially identifying groups who could benefit from targeted interventions to reduce antibiotic consumption in primary care.

## Methods

### Setting and data sources

Two extracts of the NI Enhanced Prescribing Database (EPD) were received from the NI regional data warehouse. The EPD is a regional system containing information about all prescriptions dispensed by community pharmacies in NI and is described in greater detail in other work [15].

The first extract was an aggregated dataset containing the annual quantity of antimicrobials/antibiotics (British National Formulary chapter 5.1) prescribed to each individual in NI in defined daily doses (DDDs) for all NI GP practices from 2010 to 2019. Northern Ireland Statistics and Research Agency (NISRA) mid-year population estimates (www.nisra.gov.uk) were added to the aggregated dataset to enable calculation of annual prescribing rates.

The second extract was an anonymised, individual-level dataset containing sociodemographic and catheter use information for every person registered with a GP practice in NI at 01 January 2019 and total number of DDDs prescribed to them during the year 2019. The dataset was derived via a linkage process to join the EPD to the regional GP register using the patient health and care number (HCN). HCN was available for 100% of patients in the GP register and approximately 85–90% of prescriptions in the EPD.

### Outcome variables

#### Independent outcome: annual national antibiotic prescribing rate

The annual antibiotic prescribing rate for NI (total DDDs/mid-year per 1000 population) between 2010 and 2019 was calculated from the aggregated dataset using the formula: (total DDDs/mid-year population) × 1000.

#### Individual antibiotic prescription (Yes/No) during 2019

Total individual prescribed DDDs were collapsed into a binary variable. Individuals with DDDs greater than zero were categorised as ‘Yes’, individuals with DDDs equal to zero were categorised as ‘No’.

#### Individual total prescribed antibiotics during 2019 (total number of DDDs)

A sub-analysis was then conducted on a subset of the main dataset containing only individuals who had been prescribed an antibiotic in 2019. The individual total prescribed antibiotics during 2019 (*total number of DDDs*) was the outcome for the sub-analysis.

### Risk factors for antibiotic prescribing

Sociodemographic and health-related risk factors used (in the individual-level analysis only) included; individual's age at the start of the study period, sex, Northern Ireland Multiple Deprivation Measure 2017 (NIMDM 2017) quintile based on individual's postcode (www.nisra.gov.uk), settlement band (Urban, Intermediate, Rural dwelling) based on individual's postcode (www.nisra.gov.uk) and catheter use (Yes/No) during 2019.

### Cluster-level variables (random effects)

The GP practice an individual was registered with at the start of the study period and affiliated GP federation at the start of the study period were included as cluster variables (random effects).

### Sampling and power

Given that sufficiently large samples will almost always demonstrate significant differences when using a statistical test – unless no effect whatsoever exists [16] – a simple random sample large enough to find minimal detectable differences was drawn from the individual-level, population-wide dataset. Each person appeared only once in the list. A sample size calculation was performed based on the most complicated interaction possible within the dataset (sex and deprivation quintile). A sample size of 10 000 was determined giving power of 80% to detect an odds ratio of 1.5 in the logistic regression analysis at 5% significance level. This included a design effect of two and inflation by a factor of 1.01 to account for missing data.

### Statistical analyses

The aggregated dataset was used to plot the NI annual prescribing rate (total DDDs per 1000 inhabitants per year) over time.

The individual-level analysis was carried out in two distinct phases; 1. Multilevel logistic regression analysis (with federation and practice as random effects, with the latter nested in the former) to identify factors associated with the prescription (Yes/No) of any antibiotic; 2. Multilevel linear regression analysis (with the same random effects) to identify factors associated with the quantity of antibiotic prescribed (measured in DDDs).

#### Multi-level logistic regression analysis

Univariable multilevel logistic regression analysis was conducted to ascertain the association between the log-odds of receiving an antibiotic (DDDs greater than zero) and each of the risk factors. The assumption of linearity between age and antibiotic prescription was assessed by comparing models including age, and in turn, square, cubed and quartic functions of age, employing the likelihood ratio test (LR test) to compare model fit at each stage. Where non-linearity was indicated, age-groups were derived and used in place of continuous age in both univariable and multivariable analyses. Ten-year age-groups were chosen to minimise the loss of information due to categorisation.

The assumption of independence was assessed in each univariable model by including both GP practice and federation as random effects and using the approximate likelihood ratio test to compare the mixed-effects against fixed effects models to assess if the variance components for each of the random effects were significantly different from zero. Due to the multitude of evidence suggesting a difference in antibiotic prescribing to males and females, consideration in both univariable and multivariable analyses was given to interactions between sex and all other covariates; sex and deprivation, sex and settlement band, sex and catheter use, and sex and age group. All risk factors and interactions that had been significant (at the 0.05 level) in the univariable analysis were entered into a multivariable logistic regression analysis in a forward-stepwise fashion. Sex and age group were retained in all multivariable models regardless. Confounding was considered at each step of the model-building process, defined as a change of more than 10% in the odds ratio (OR) of parameters still in the model with the addition or removal of the confounder. Interactions were considered during multivariable analysis with main effects entered initially, then removed and replaced by the interaction term. The LR test and Akaike information criterion (AIC) were used to compare fit.

#### Multi-level linear regression analysis

Secondly, a sub-analysis was conducted focusing only on individuals who received an antibiotic during the year 2019 (DDDs greater than zero). Univariable and multivariable analyses were conducted using the same approach described above with total number of DDDs prescribed during 2019 as the outcome variable. Confounding was defined as a change of more than 10% in the regression coefficient of parameters still in the model with the addition or removal of the confounder. Post-regression analysis using residual-versus-fitted and quantile-quantile plots suggested deviation from normality, non-constant variance and a trend in residuals. To overcome this, a log-transformation (natural logarithm) was performed on the outcome variable and the linear analysis repeated using the log-transformed outcome. Again, the residuals from the final model violated the assumption of normality, albeit not so strongly. It was also noted that the fit of the linear regression model was generally poor.

#### Finite Mixture Modelling (FMM)

To improve model fit and adherence to model assumptions, a finite mixture modelling (FMM) approach [17] was conducted with the log transformed DDDs outcome. The number of suspected latent classes was decided *a priori* and models were estimated with seven, down to two classes. Convergence was achieved for the five and two class models and they were compared for fit against the final model from the simple linear regression analysis using the AIC. To further improve fit, risk factor variables that determined membership of each class were modelled, each entered into the model in a forward stepwise procedure and the AIC used to compare. The model with deprivation included did not converge. Once the final model had been estimated, the assumption of clustering was tested using the AIC to compare models with and without the cluster variables.

Analysis of the annual NI antibiotic prescribing rate was conducted using R version 4.1.0 for Windows [18], all other statistical analyses were conducted using Stata/IC version 16.1 for Windows [19].

#### Ethics

Ethical approval was not necessary. No personally identifiable information was processed during this study, all data were anonymised prior to receipt.

## Results

### Trends in primary care antibiotic prescribing

**Fig. 1.**
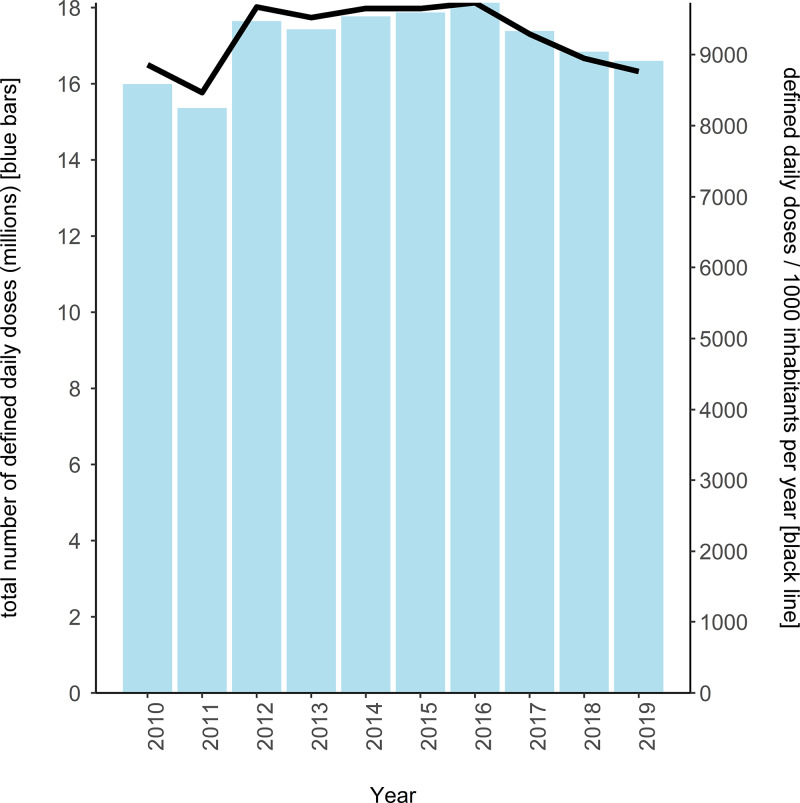
Total number of defined daily doses (DDDs) and prescribing rate (defined daily doses per 1000 inhabitants per year), for all antibiotics in primary care, Northern Ireland, 2010–2019. Blue bars indicate total number of defined daily doses in millions; black line indicates the prescribing rate per 1000 inhabitants per year.

Between 2010 and 2019 the average prescribing rate was 9258.66 DDDs per 1000 inhabitants, ranging from 8467.84 DDDs per 1000 inhabitants in 2011 to 9735.43 DDDs per 1000 inhabitants in 2016. A clear downward trend was observed since 2016, when the O'Neill review was released (Fig. 1).

### Individual-level analysis

#### Study population

Age among the 10 000 individuals sampled ranged from zero to 102 years with a median of 38 years (interquartile range (IQR) = 19–57). Fifty-four per cent of the sample was male and 99.3% of individuals had not received a catheter during 2019. There was a fairly even split across deprivation quintiles. The majority of individuals in the sample lived in an intermediate settlement band (72.9%, *n* = 5729) (Table 1).

**Table 1.** The unadjusted odds of being prescribed an antibiotic by sociodemographic factor, settlement band and catheter status in Northern Ireland, January–December 2019

	*n*	Prescribed at least one antibiotic during 2019 *n* (%)	Not prescribed an antibiotic during 2019 *n* (%)	OR	95% CI	GP Practice variance component (*n* = 330)	GP Federation variance component (*n* = 17)
Total sample	10 000	3020 (30.2%)	6980 (69.8%)				
Age Group							
0–10	1436	470 (32.7%)	966 (67.3%)	1.54	1.30–1.82		
11–20	1211	307 (25.4%)	904 (74.6%)	1.07	0.89–1.28		
21–30	1264	312 (24.7%)	952 (75.3%)	1.03	0.86–1.24		
31–40	1362	321 (23.6%)	1041 (76.4%)	0.97	0.81–1.16		
41–50	1312	317 (24.2%)	995 (75.8%)	Ref			
51–60	1383	415 (30.0%)	968 (70.0%)	1.35	1.14–1.61		
61–70	954	350 (36.7%)	604 (63.3%)	1.82	1.51–2.19		
71–80	712	313 (44.0%)	399 (56.0%)	2.49	2.05–3.03		
81–90	320	186 (58.1%)	134 (41.9%)	4.40	3.40–5.69		
91+	46	29 (63.0%)	17 (37.0%)	5.49	2.96–10.17	0.032	0.004
Sex							
Male	5043	1186 (23.5%)	3857 (76.5%)	Ref			
Female	4957	1834 (37.0%)	3123 (63.0%)	1.93	1.77–2.11	0.045	0.003
Deprivation (*n* = 9939)		3000 (30.2%)	6939 (69.8%)				
Most Deprived	1964	591 (30.1%)	1373 (69.9%)	Ref			
2	2083	623 (29.9%)	1460 (70.1%)	0.99	0.87–1.15		
3	2052	640 (31.2%)	1412 (68.8%)	1.07	0.93–1.23		
4	2054	609 (29.7%)	1445 (70.3%)	0.99	0.86–1.15		
Least Deprived	1786	537 (30.1%)	1249 (69.9%)	1.02	0.88–1.19	0.037	0.004
2015 Settlement Band (*n* = 9939)		3000 (30.2%)	6939 (69.8%)				
Urban	1514	414 (27.3%)	1100 (72.7%)	Ref			
Intermediate	5729	1770 (30.9%)	3959 (69.1%)	1.19	1.03–1.37		
Rural Dwelling	2696	816 (30.3%)	1880 (69.7%)	1.16	0.99–1.36	0.036	0.003
Catheter Status							
No catheter	9972	2997 (30.1%)	6975 (69.9%)	Ref			
Had a catheter	28	23 (82.1%)	5 (17.9%)	10.74	4.06–28.42	0.037	0.003

#### Multilevel logistic regression analysis

Table 1 shows the results of the univariable multilevel logistic regression analysis. Approximately 30% of the sample (*n* = 3020) were prescribed an antibiotic during 2019.

Females displayed almost double the odds of being prescribed an antibiotic compared to males (OR 1.93, 1.77–2.11, *P* < 0.001). Compared to people aged 41–50 years (the reference group) the odds of being prescribed an antibiotic were higher among those in the youngest age group (0–10 years) but similar among those aged between 11 and 40 years. The odds of being prescribed an antibiotic then increased exponentially from the age of 51 onwards.

Deprivation was not significantly associated with being prescribed an antibiotic in the univariable analysis, with little difference in the odds observed across deprivation quintiles. Settlement band was almost significantly associated at the 0.05 level (*P* = 0.06). Compared to those living in an urban environment, odds of being prescribed an antibiotic were higher for those living in an intermediate area, but no difference was observed among those living in a rural area. People who used a catheter at least once during the study period displayed more than ten times the odds of being prescribed an antibiotic than those without a catheter (OR 10.74, 4.06–28.42, *P* < 0.001).

Table 2 displays the results of the multivariable logistic regression modelling. The final multilevel logistic regression model included an interaction between age and sex, settlement band and catheter use (fixed effects) and GP practice and federation (random effects). Overall the model was significantly associated with antibiotic prescribing (*χ*^2^ = 496.89, *P* < 0.001) and was better than the fixed effects logistic model (LR test *P* < 0.01).

**Table 2.** The adjusted odds of being prescribed an antibiotic by sociodemographic factor, settlement band and catheter status in Northern Ireland, January–December 2019

	*n*	Prescribed at least one antibiotic during 2019 *n* (%)	aOR^a^	95% CI	ICC (95% CI)
Total sample	10 000	3020 (30.2%)			
Age & Sex					
*Males*					
0–10	713	218 (30.6%)	2.27	1.75–2.94	
11–20	646	138 (21.4%)	1.38	1.05–1.83	
21–30	621	97 (15.6%)	0.95	0.70–1.27	
31–40	710	104 (14.7%)	0.88	0.66–1.18	
41–50	695	112 (16.1%)	Ref		
51–60	711	165 (23.2%)	1.55	1.18–2.03	
61–70	478	146 (30.5%)	2.22	1.67–2.95	
71–80	335	134 (40.0%)	3.41	2.25–4.61	
81–90	121	64 (52.9%)	5.38	3.53–8.20	
91+	13	8 (61.5%)	8.40	2.67–26.42	
*Females*					
0–10	723	252 (34.9%)	2.57	1.97–3.34	
11–20	565	169 (29.9%)	2.22	1.69–2.92	
21–30	643	215 (33.4%)	2.59	1.99–3.37	
31–40	652	217 (33.3%)	2.58	1.98–3.35	
41–50	617	205 (33.2%)	2.57	1.97–3.34	
51–60	672	250 (37.2%)	3.06	2.36–3.96	
61–70	476	204 (42.9%)	3.87	2.94–5.09	
71–80	377	179 (47.5%)	4.71	3.53–6.29	
81–90	199	122 (61.3%)	8.05	5.65–11.46	
91+	33	21 (63.6%)	8.55	4.03–18.13	
2015 Settlement Band (*n* = 9939)					
Urban	1514	414 (27.3%)	Ref		
Intermediate	5729	1770 (30.9%)	1.15	0.99–1.33	
Rural Dwelling	2696	816 (30.3%)	1.12	0.95–1.32	
Catheter Status					
No catheter	9972	2997 (30.1%)	Ref		
Had a catheter	28	23 (82.1%)	6.82	2.50–18.64	
Random Effects			Variance estimate		
GP Practice	330		0.037	0.015–0.092	0.012 (0.005–0.028)
GP Federation	17		0.004	0.000–0.046	0.001 (0.000–0.014)

a Adjusted for all variables in the table and for clustering at GP practice and GP federation levels.

The interaction between age and sex was significantly associated with being prescribed an antibiotic (*P* < 0.001) in the final model. With the exception of males aged 21–30 and 31–40 years, the adjusted odds of being prescribed an antibiotic remained higher among males in all other age groups. Compared to males aged 41–50 years, the adjusted odds of being prescribed an antibiotic remained higher for females in all age groups. Settlement band was not significantly associated with antibiotic prescribing in the multivariable analysis but was retained as it improved the AIC of the final model. Having a catheter remained significantly associated with being prescribed an antibiotic (*P* < 0.001). Those who used a catheter at least once during the study period displayed almost seven times the odds of being prescribed an antibiotic than those who did not (aOR 6.82, 2.50–18.64). The final model also included both random effects. Of the total variation in the odds of antibiotic prescribing, 1.24% was attributable to the variation between GP practices (intraclass correlation coefficient (ICC) = 0.0124) and 0.12% to the variation between GP federations (ICC = 0.0012).

#### Linear regression analysis

The results of the sub-analysis of the quantity of antibiotics measured in log-transformed DDDs prescribed to those who received an antibiotic during 2019 (*n* = 3020) are shown in Tables 3 and 4. The majority of those who received an antibiotic were female (60.7%) with age ranging from 0 to 102 years, median age was 44 years (interquartile range (IQR) = 20–64 years). Ninety-nine per cent of individuals had not received a catheter during 2019 and there was a fairly even split across deprivation quintiles. The largest proportion of individuals who had received an antibiotic lived in an intermediate settlement band (59%, *n* = 1770).

**Table 3.** The unadjusted variation in log transformed DDDs prescribed for individuals registered with a GP practice and prescribed an antibiotic, by sociodemographic factor, settlement band and catheter status in Northern Ireland, January–December 2019

	*n (%)*	Unadjusted coefficient	95% CI	*P*-value
Total sample	3020			
Age Group				
0–10	470 (15.6%)	−0.96	−1.08 to −0.83	
11–20	307 (10.2%)	−0.15	−0.29 to −0.01	
21–30	312 (10.3%)	−0.21	−0.35 to −0.06	
31–40	321 (10.6%)	−0.21	−0.34 to −0.07	
41–50	317 (10.5%)	Ref		
51–60	415 (13.7%)	0.00	−0.13 to 0.13	
61–70	350 (11.6%)	0.13	0.00–0.27	
71–80	313 (10.4%)	0.24	0.11–0.38	
81–90	186 (6.2%)	0.08	−0.08 to 0.24	
91+	29 (0.96%)	−0.02	−0.35 to 0.31	0.000
Sex				
Male	1186 (39.3%)	Ref		
Female	1834 (60.7%)	−0.05	−0.12 to 0.02	0.137
Deprivation (*n* = 3000)				
Most Deprived	591 (19.7%)	Ref		
2	623 (20.8%)	0.04	−0.07 to 0.14	
3	640 (21.3%)	−0.03	−0.13 to 0.08	
4	609 (20.3%)	0.03	−0.07 to 0.14	
Least Deprived	537 (17.9%)	−0.04	−0.15 to 0.08	0.567
2015 Settlement Band (*n* = 3000)				
Urban	414 (13.8%)	Ref		
Intermediate	1770 (59%)	−0.08	−0.18 to 0.02	
Rural Dwelling	816 (27.2%)	−0.04	−0.15 to 0.07	0.236
Catheter Status				
No catheter	2997 (99.2%)	Ref		
Had a catheter	23 (0.76%)	0.76	0.37–1.15	0.000

**Table 4.** The adjusted variation in log transformed DDDs prescribed for individuals registered with a GP practice and prescribed an antibiotic per class, by sociodemographic factor, settlement band and catheter status in Northern Ireland, January–December 2019

	Low antibiotic use/DDD (mean log DDDs = 2.1, class membership probability = 20%)			High antibiotic use/DDD (mean log DDDs = 2.7, class membership probability = 80%)			LR test *P*-value
	Adjusted coefficient^a^	95% CI	Wald *P*-value	Adjusted coefficient^a^	95% CI	Wald *P*-value	
*Total Sample*							
Age Group							
0–10	−0.53	−0.65 to −0.40		−1.08	−1.24 to −0.92		
11–20	−0.04	−0.20 to 0.12		−0.15	−0.34 to 0.30		
21–30	−0.06	−0.19 to 0.07		−0.17	−0.35 to 0.01		
31–40	−0.02	−0.14 to 0.10		−0.16	−0.34 to 0.02		
41–50	Ref			Ref			
51–60	0.02	−0.12 to 0.16		0.01	−0.16 to 0.18		
61–70	0.21	0.05–0.38		0.09	−0.08 to 0.26		
71–80	−0.08	−0.23 to 0.06		0.23	0.05–0.40		
81–90	−0.18	−0.35 to −0.01		0.04	−0.16 to 0.24		
91+	−0.17	−0.52 to 0.19		−0.08	−0.50 to 0.34		0.000
Sex							
Male	Ref						
Female	0.01	−0.05 to 0.07	0.740	−0.24	−0.33 to −0.15	0.000	–
Deprivation (*n* = 3000)							
Most Deprived	Ref			Ref			
2	0.01	−0.08 to 0.90		0.06	−0.70 to 0.18		
3	0.01	−0.07 to 0.10		−0.03	−0.16 to 0.09		
4	0.05	−0.04 to 0.13		0.02	−0.11 to 0.15		
Least Deprived	0.04	−0.05 to 0.13	0.817	−0.07	−0.20 to 0.06	0.333	–
2015 Settlement Band (*n* = 3000)							
Urban	Ref			Ref			
Intermediate	−0.05	−0.08 to 0.09		−0.15	−0.27 to −0.02		
Rural Dwelling	−0.02	−0.12 to 0.07	–	−0.11	−0.25 to 0.04	–	0.128
Catheter Status							
No catheter	Ref			Ref			
Had a catheter	0.64	0.47–0.82	0.000	0.80	0.25–1.34	0.004	–

a Adjusted for all variables in the table.

*Note:* Wald *P*-values reported where likelihood ratio test *P*-values were not available due to model non-convergence.

Table 3 displays the results of the univariable analysis. There was a significant association between age group and the variation in log DDDs (*P* < 0.001). Compared to people aged 41–50 years; a smaller quantity of antibiotics was prescribed to people in the younger age groups. Number of log DDDs prescribed to those aged 51–60 years was similar to those aged 41–50 while an increase was observed among those who were older, with the exception of those aged more than 90 years. There was no association between sex and number of log DDDs prescribed (*P* = 0.137). Deprivation (*P* = 0.567) and settlement band (*P* = 0.236) were also not significantly associated in the univariable analysis.

Those who had used a catheter at least once during the year were prescribed significantly more than those without a catheter (0.76 greater on the log scale (0.76, 0.37–1.15, *P* < 0.001). None of the interactions investigated (Sex and Deprivation, Sex and Catheter use and Sex and Age group) were found to be significant in the univariable analysis and the variation in log DDDs did not differ between GP practices or federations, thus the univariable models were simplified to contain only fixed effects.

#### Finite mixture modelling

Table 4 shows the results of the FMM analysis. Models assuming five and two classes achieved convergence and were compared using their AICs, with the two-class model judged to be better (lower AIC). The two classes within the final model effectively corresponded to ‘low’ and ‘high’ log DDDs. The mean for individuals in class 1 (‘low antibiotic use/DDD’) was 2.1 log DDDs while the mean in class 2 (‘high antibiotic use/DDD’) was 2.7 log DDDs. The probability of low antibiotic use/DDD membership was 20% and so the probability of high antibiotic use/DDD membership was 80%. Class membership was determined by: age group, sex, settlement band and catheter use. Females had significantly higher odds of being in the high antibiotic use/DDD class than males (OR 2.70, 95% CI 1.88–3.89, *P* < 0.001) and those aged 31–40 years had significantly lower odds (of being in the high antibiotic use/DDD class) than those aged 41–50 years (OR 0.46, 0.24–0.91, *P* < 0.05). Comparison of clustered and non-clustered models indicated that assuming clustering did not improve model fit and so the simple fixed-effects model was chosen.

Fixed-effect risk factors for each class included in the final model were: age group, sex, deprivation, settlement band and catheter use. Age was significantly associated with log DDDs (LR test *P* < 0.001). In the low antibiotic use/DDD class, a significant decrease in log DDDs was observed (compared to those aged 41–50 years) for those aged 0–10 years (decrease of 0.53 on the log scale (−0.53, −0.65 to −0.40)) and 81–90 years (decrease of 0.18 on the log scale (−0.18, −0.35 to −0.01)), while a significant increase was observed among those aged 61–70 years (increase of 0.21 on the log scale (0.21, 0.05–0.38)). In the high antibiotic use/DDD class, compared to those aged 41–50 years, a significant decrease was again observed among those aged 0–10 years (decrease of 1.08 on the log scale (−1.08, −1.24 to −0.92)) with an increase observed in the 71–80 age group (increase of 0.23 on the log scale (0.23, 0.05–0.40)).

There was no significant association between sex and log DDDs in the low antibiotic use/DDD class but a significant association was observed in the high antibiotic use/DDD class (Wald *P* < 0.001). Females in the high antibiotic use/DDD class were associated with a decrease in log DDDs compared to males (decrease of 0.24 on the log scale (−0.24, −0.33 to −0.15)). Deprivation was not significantly associated with log DDDs in either the high or low antibiotic use/DDD class but was retained in the final model as it confounded the relationship between settlement band and log DDDs. Settlement band was also not significantly associated with log-DDDs but was retained in the final model as it improved the model AIC. Catheter use was significantly associated with log-DDDs for individuals in both the low (Wald *P* < 0.001) and high antibiotic use/DDD classes (Wald *P* < 0.005). In the low antibiotic use/DDD class, those who had used a catheter at least once during 2019 were associated with an increase in log DDDs compared with those who had no catheter (increase of 0.64 on the log scale (0.64, 0.47–0.82)). A similar trend was observed for individuals in the high antibiotic use/DDD class (increase of 0.80 on the log scale (0.80, 0.25–1.34)).

Finally, comparison of the final FMM model against the multiple linear regression model using the AIC showed that while fit was still relatively poor, it improved using the finite mixture model.

## Discussion

We used routine administrative data to describe the time-series of trends in primary care prescribing in Northern Ireland and provide insight into who receives antibiotics in primary care, taking account of the role of GP practices and federations. Antibiotic prescribing in NI primary care is generally reducing. Females were more likely to receive an antibiotic than males and at a younger age while, similar to previous studies, younger and older people were generally more likely to receive an antibiotic than those in the middle age groups. Among those who were prescribed, however, males received a slightly larger quantity of antibiotics than females. Surprisingly, deprivation and urban/rural settlement were not associated with either the odds of antibiotic prescribing or the quantity prescribed. Catheter use was strongly associated with both odds of prescribing and the amount prescribed, and variation was observed between GP practices and to a lesser degree, between GP federations.

At this point in time, it is not clear what the short to medium-term impact of the COVID-19 pandemic will be on antimicrobial consumption in primary care, but it is clear that to reduce the threat of AMR to future population health, antimicrobial stewardship should be strengthened and our use of antibiotics monitored. The general trend in prescription of antibiotics in Northern Ireland primary care to 2019 was downward. Since the O'Neill Review of Antimicrobial Resistance in 2016 [1], reduction of antibiotic consumption (in both primary and secondary care) has been high on the NI Department of Health's public health agenda and has formed the backbone of consecutive AMR action plans [20, 21]. Several interventions have been implemented to drive down prescribing specifically in primary care, including piloting a social norm feedback intervention [22], rolling out TARGET antibiotics toolkit [23] workshops for primary healthcare staff, pilot of a decision-aid [24, 25] to reduce inappropriate antibiotics for UTIs in care homes [26], and piloting the implementation of CRP point-of-care testing in NI community pharmacies and GP practices with the aim of reducing inappropriate GP presentations [2].

However, there is still room for improvement. While studies from some other countries have shown higher prevalence of primary care antibiotic prescribing [27, 28], within the European community antibiotic prescribing in the UK was around mid-table in 2019 [29].

Findings from the individual-level analysis largely support the findings of previous studies in other countries. Age, sex and catheter use were the main predictors of both being prescribed an antibiotic and the quantity of antibiotic prescribed (in DDDs). As in previous studies [7, 30], females were more likely to receive antibiotics than males, and at a younger age. These findings may at least partly be explained by gender differences in health-seeking behaviour. Previous work suggests differences in prescribing to males and females approximate the differences in health-seeking behaviour, with men as much as 80% [31] less likely to consult primary care. Reduced and delayed help-seeking among males is a well-established phenomenon [31, 32] partly attributable to female reproductive-related consultations [32] and conditions that more commonly affect adult females such as UTIs, which also account for a significant proportion of antibiotic prescriptions in primary care [31].

Other possible explanations for this gender gap however include a mixture of psychological and socio-demographic factors, and some features of the health system itself [33]. Cognitive factors include attitudes of masculinity [34, 35] and endorsement of a view that men should be reluctant to seek help unless perceived as a means to preserve another enactment of masculinity such as maintaining sexual performance [36]. Stigma associated with help-seeking perpetuates this reluctance [35]. Interestingly however, while females were more likely to be prescribed an antibiotic, males generally received a greater quantity once prescribed. This could be related to delayed help-seeking among males, leading to more severe illness at presentation [34, 37]. UTIs diagnosed among males are usually classed as ‘complicated’ for physiological reasons, also requiring a longer course of treatment.

Having had a catheter at some stage during the year was strongly associated with both antibiotic prescription and the amount of antibiotic prescribed. Due to the low prevalence of catheter use in the population, there was uncertainty around the estimates of antibiotic consumption in both analyses, reflected in the width of the confidence intervals. Having a catheter in-situ greatly increases the likelihood of a urinary tract infection (UTI) requiring antibiotic treatment [13] and may also be prescribed prophylactically prior to catheter removal for patients with a history of urinary tract infection, although NICE does not support this practice [13].

Interestingly, no association between deprivation and antibiotic prescription or quantity of antibiotics prescribed was found in either analysis. The link between socioeconomic deprivation and antibiotic prescribing has been well established and reported in several previous studies [7, 38] although at least one other study has replicated the results found here [39]. There may be a number of explanations for this finding. Firstly, unlike England, antibiotics in Northern Ireland are free of charge for the entire population and therefore widely accessible. Also, those in the most and least deprived quintiles on average live closest to a GP practice [40]. Secondly, while people living in areas of higher socioeconomic deprivation often incur greater incidence of infections requiring antibiotic treatment [41, 42], they may refrain from engaging with healthcare and have a greater propensity for non-adherence [43]. This *‘unmet need’* has been similarly observed in at least one other NI-based study looking at prescribing of obesity medication [44]. Finally, those living in more affluent areas may have an increased perception of risk [45] and more confidence in their ability to seek health information [46] leading to increased demand for antibiotics. Indeed, a recent study in NI reported that, while higher deprivation was associated with increased rates of overall prescribing, lower deprivation was associated with a higher mean cost of prescription [38]. These competing influences may in effect cancel each other out, dampening the socioeconomic gradient usually observed with most health outcomes.

No association was found between settlement band and either of the prescribing outcomes, although its inclusion in the models did improve model fit. Research around urban/rural settlement and antibiotic prescribing has so far produced ambiguous results with evidence supporting associations in both directions [10, 47]. Given that urban-based practices in NI are generally in closer proximity to their catchment population [48] thus improving access [49], the finding here was unexpected and the reasons unclear.

The use of multi-level analysis enabled the ability to account for the role of GP practices and federations in antibiotic prescribing by adjusting for the hierarchical nature of the data. This adjustment may in itself account for some of the unusual findings including the lack of associations with deprivation and settlement band. Echoing previous work in England [50] and Wales [51], variation across GP practices and GP federations was observed, although to a smaller, but not negligible extent. Some of this variation is likely attributable to unmeasured differences in the case-mix of GP practice catchment populations. It is also likely, however, that some practices may have higher rates of inappropriate prescribing [52], the reasons for which are complex and may include characteristics of the patient, practice and the prescriber [3]. It was not possible to measure prescriber characteristics in this study. While the amount of between-GP and federation variation observed in this study was small, it was not negligible and any room from improving antimicrobial stewardship in primary care should be utilised. These findings provide some further evidence of the need for close partnership working between health services and GP practices and implementation of a joint patient and practice-wide approach [53,54] to improving antimicrobial stewardship in primary care. Work on this continues in NI [2, 22].

### Strengths and limitations

This analysis used a national primary care prescribing dataset to look at antibiotic use and associated sociodemographic risk factors. While clinical information wasn't directly available, catheter use was considered as a proxy measure. Catheter use is a known risk factor for infection [13] and so it's inclusion in the models allowed some level of adjustment for patient comorbidities. The use of a multilevel modelling approach enabled adjustment for the hierarchical nature of the data, improving the accuracy of the estimates in the regression models.

There were also a number of limitations. The cross-sectional study design limits the ability to directly observe causal relationships and makes it difficult to generalise the findings to time-periods beyond the scope of the study. This may be particularly important in the wake of the COVID-19 pandemic. Limited information about GP practices and their catchment populations also made it challenging to fully explain the observed between-practice and between-federation variation, while a lack of clinical information made it impossible to measure appropriateness of antibiotic prescribing.

Using a national-level prescribing dataset it was possible to control for known confounders such as age and sex. Data were also available for catheter use, which is a known risk factor for urinary tract infections. However, in the general population the majority of antibiotics are given for respiratory tract infections and using the prescribing dataset it was not possible to measure other patient comorbidities that might increase the risk of infection, and therefore the need for antibiotics. Therefore, residual confounding due to unmeasured covariates is likely.

It is also important to note the generally poor fit of the mixture models in the analysis of log DDDs. This is again likely explained by the absence of important covariates. While the mixture models showed improved fit over the simple linear regression model, they still accounted for only a small proportion of the overall variation in log DDDs.

This study focused only on prescription of all antibiotics, further research around the trends and factors associated with prescription of specific antibiotics, such those prescribed for UTIs which account for a large proportion of prescriptions may be provide important insight and highlight further opportunities for improving antimicrobial stewardship in NI primary care.

Finally the findings are produced in the specific context of Northern Ireland's population and its health system, so the findings may not be applicable in other contexts.

## Conclusion

Our study provides useful information for policy-makers, public health authorities, healthcare systems and primary care prescribers. It also highlights the importance of accounting for clustering within hierarchical data. Overall, the findings suggest females, those in the older age-groups and parents of young children should be the focus of campaigns to reduce antibiotic prescribing in primary care. Some attention should also be paid to ensuring proper management of people with catheters and other comorbidities in line with current guidance. Finally, any campaign to reduce prescribing in primary care should involve GP practices themselves, perhaps in the form of engagement through the NI GP federations.

## Supplementary material

For supplementary material accompanying this paper visit http://doi.org/10.1017/S0950268822000644. click here to view supplementary material

## Availability of data and materials

The corresponding author (CN) has access to the data; however any 3rd party access would be contingent on data access agreements with the Northern Ireland Honest Broker Service [55].
