# Supplementary material for: Examining the association between socio-demographic factors, catheter use and antibiotic prescribing in Northern Ireland primary care: a cross-sectional multilevel analysis
Source: Epidemiol Infect. 2022 Apr 21;150:e92. doi: 10.1017/S0950268822000644 (PMC9102062; doi:10.1017/S0950268822000644)
Supplement: Supplementary file 1 [file S0950268822000644sup001.docx]

**Supplementary table 1.** Antibiotic prescribing in primary care, Northern Ireland, for all antibiotics (BNF 5.1) expressed in number of defined daily doses (DDDs) and the NI mid-year population estimate (from [www.nisra.gov.uk](http://www.nisra.gov.uk)), 2010-2019

| Year | Total number of DDDs | Mid-Year Population Estimate |
| --- | --- | --- |
| 2010 | 15993251 | 1804833 |
| 2011 | 15363353 | 1814318 |
| 2012 | 17644944 | 1823634 |
| 2013 | 17431018 | 1829725 |
| 2014 | 17771507 | 1840498 |
| 2015 | 17874447 | 1851621 |
| 2016 | 18128699 | 1862137 |
| 2017 | 17386617 | 1870834 |
| 2018 | 16841619 | 1881641 |
| 2019 | 16600872 | 1893667 |
